# Epidemiology and molecular characterization of re-emerged virulent strains of Peste des Petits Ruminants virus among sheep in Kassala State, Eastern Sudan

**DOI:** 10.1186/s13620-021-00202-5

**Published:** 2021-09-07

**Authors:** Fatima A. Saeed, Mohammed M.Gumaa, Sana A.Abdelaziz, Khalid A. Enan, Selma K. Ahmed, Mohammed O. Hussien

**Affiliations:** 1 Kassala Veterinary Research Laboratory (KVRL), Central Veterinary Research Laboratories (CVRL), Animal Resources Research Corporation (ARRC), Al Amarat, P.O. Box 237P.O. Box 8067, Khartoum, Sudan; 2 Department of Microbiology, Faculty of Veterinary Medicine, University of Khartoum, P.O. Box 32, Khartoum North, Sudan; 3 Central Laboratory, Ministry of Higher Education and Scientific Research, P.O. Box 7099, Khartoum, Sudan; 4 Central Veterinary Research Laboratory (CVRL), Animal Resources Research Corporation (ARRC), Al Amarat, P.O. Box 8067, Khartoum, Sudan

**Keywords:** Epidemiology, Molecular characterization, PPRV, Sheep, Kassala, Sudan

## Abstract

**Background:**

Peste des Petits Ruminants (PPR) is a severe contagious viral disease, which mainly affects small ruminants. PPR is caused by a *Morbillivirus* that belongs to the family *Paramyxoviridae*. In this study 12 suspected PPR outbreaks among sheep and goats were investigated in four localities in Kassala State, Eastern Sudan, during 2015—2017. The causative agent was confirmed by a Sandwich Enzyme-Linked Immunosorbent Assay (sELISA), and a Reverse Transcription Polymerase Chain Reaction (RT-PCR) targeting a partial sequence of nucleocapsid protein gene (N- gene) and a partial sequence of fusion protein gene (F- gene). Sequencing and phylogenetic analysis were carried out on six N- gene based RT-PCR products selected from two outbreaks occurred on border and inner localities of Kassala State to determine the circulating lineages of PPRV strains. Identity percentages were determined between isolates in this study and previous Sudanese, and other (African and Asian) isolates which clustered along with them.

**Results:**

Out of 30 samples, 22 (73.3%) were positive using sandwich ELISA. From 22 s ELISA positive samples, 17 (77.3%) were positive by Ngene based RT-PCR and only 7(43.8%) out of 16 positive samples by N gene based RT-PCR were positive using Fgene based RT-PCR. The sequencing and phylogenetic analysis confirmed involvement of the lineage IV of PPRV in outbreaks among small ruminants in Kassala State and high identity percentage between our isolates and previous Sudanese and other (African and Asian) isolates.

**Conclusions:**

The present study demonstrates that genetic relationship between PPRV strains circulating in sheep in Kassala State, Eastern Sudan, and PPRV strains characterized as lineage IV in neighboring African countries such as Eretria,Ethiopia, Egypt, and other Asian countries

## Background

Peste des Petits Ruminants (PPR) is a severe contagious viral disease which mainly affects small ruminants [1]. There are several reports of PPR occurring in other wild species, particularly in captive wild ungulates [2]. The disease caused by a virus belongs to the genus *Morbillivirus* of the family *Paramyxoviridae* [3] which grouped along with the Rinderpest virus in cattle, Measles virus in human, and Canine distemper virus in pets and wild animals species belonging to Canidae, Mustelidae, Procynonidae, Ailuridae, Elephantidae, and large Felidae [4]. PPR is characterized by depression, fever, discharges from eyes and nose, mouth sores, disturbed breathing, cough, diarrhea, and death [5]. PPRV has a single serotype which has been grouped into four distinct lineages were I, II, III, and IV based on sequence comparison of partial sequences of Fgene [6], Ngene [7], or Hgene [8]. All four lineages have been detected in Africa [9]. In Sudan, the first outbreaks of PPR were originally diagnosed as Rinderpest in 1971 and later confirmed to be PPR [10, 11], and then outbreaks continued to be reported all over Sudan. The annual vaccination program against PPR in Sudan using an attenuated vaccine (Strain 75/1) has been established in 2002. Since then, a plan to control PPRV was launched, but organized vaccination campaigns were not well performed [12, 13].

**Fig. 1:**
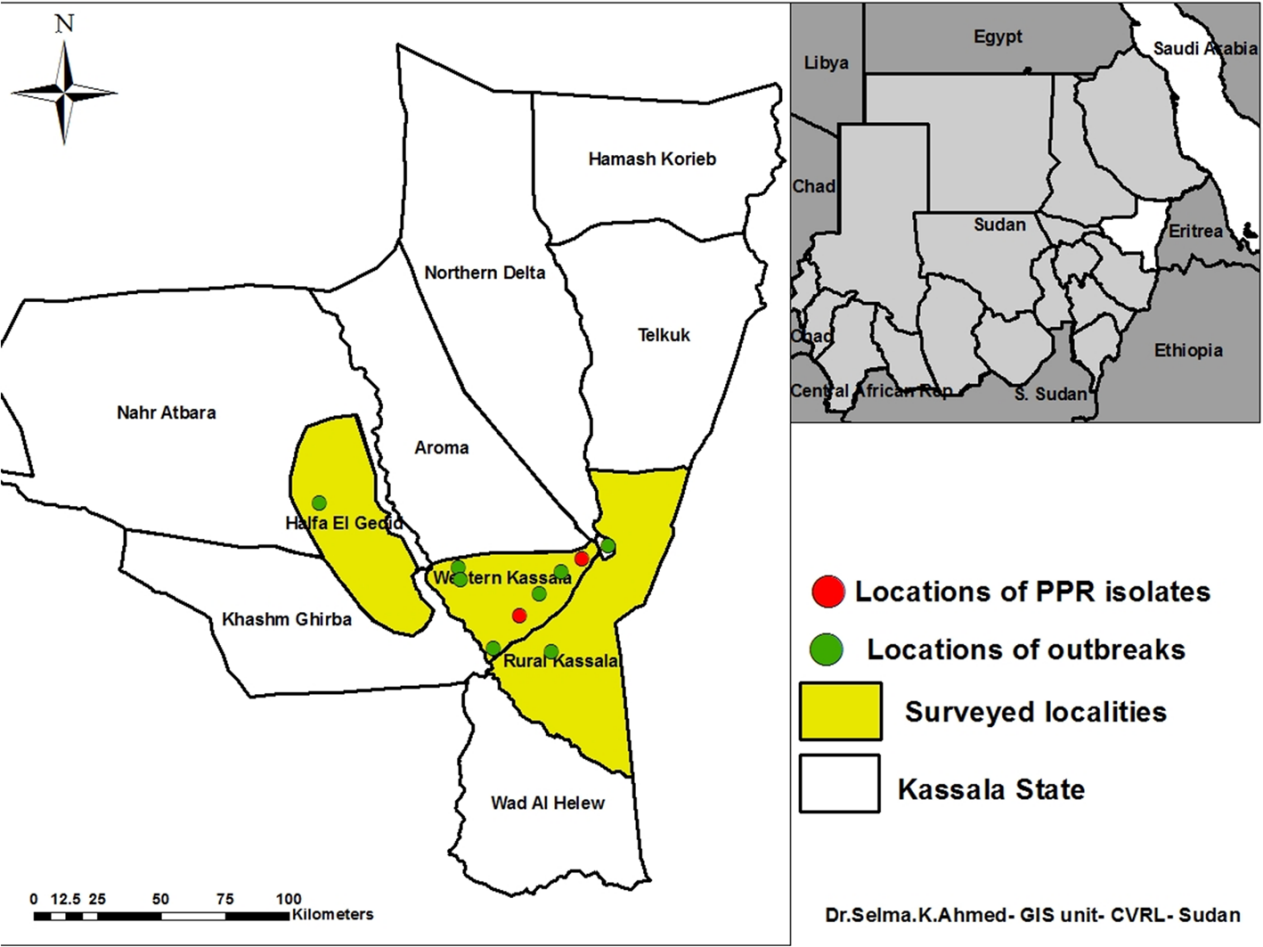
Kassala State map showed surveyed localities (yellow color), locations of outbreaks (green circle) and location of PPR isolates (red circle)

Kassala State is located in Eastern Sudan and shares porous international boundaries with Eritrea, Ethiopia, and other Sudanese States (Fig. 1). The virus was isolated from infected camels in a severe outbreak in Kassala State during August—October 2004 [14]. In last years, PPR had become endemic, and outbreaks were occurred throughout year involving different species, but virulent strains of the virus had been emerged from time to time causes a huge loss. Also PPR was investigated in four Sudanese states including Northern State, River Nile State, Khartoum State, and Gezira State during 2016–2017 found that the prevalence was higher than previously documented [15]. Antibodies against PPRV were detected in cattle imported from Sudan to Egypt [16]. Prevalence and presence of PPR viral antigen were investigated in pneumonic lungs of apparently healthy camels at Tambul slaughter house, Gezira State, Central Sudan, demonstrating the exposure and probable sub-clinical infection in camels [17].

The present study reported a confirmed molecular characterization of the virulent virus strain among sheep in Kassala State, and its comparison with available sequences from GenBank database provides valuable information about epidemiology of PPR in Sudan.

## Methods

### Outbreaks areas

Kassala State is a border state and major area of animals’ exportation. The state is divided into eleven localities. In this study, we have investigated the disease outbreaks in four localities which are rural Kassala, rural West Kassala, rural New Halfa, and Kassala (Fig. 1). The selected localities had records of suspected PPR outbreaks in which some of them are more severe in a period between October, 2015 and February, 2017. In 2015, there was an outbreak in rural new Halfa locality with clinical signs of fever, diarrhea, and abortion. In 2016, outbreaks were located in rural Kassala and rural West Kassala. The main signs were fever, diarrhea, lacrimation and oral erosions. Severe suspected PPR outbreaks among sheep flocks were occurred in February, 2017 in different villages of Kassala State with similar signs. Two of these villages (Noora and Kalhot) were visited after a few hours of onset of the disease symptoms. Noora village was located in rural West Kassala Locality representing the inner site of Kassala State whereas Kalhot village in rural Kassala Locality representing the border site of Kassala State. In these two outbreaks, the animals aged of 6–7 months from *Garaj* and *Gash* breeds were affected. Clinical signs are demonstrated in Table 1. Detailed information about outbreaks from 2015–2017 is presented in Table 1.

**Table 1:** Data of 12 suspected PPR outbreaks investigated in Kassala State

Locality and Area	GPS data	flock size	infected animals	dead animals	Clinical signs	collected samples	Type of samples
Rural New Halfa (faras village 6/10/2015)	N:15.2578° E:35.40416°	150 goats	11	3	Fever, abortion, diarrhea, oral erosions,	2	(O.S,R.S)
Rural Kassala (Jabal eilak village 1/9/2016)	N:15.108060° E:36.269701°	40 sheep	7	1	fever, diarrhea, lacrimation, oral erosions, nasal discharge	1	(N.S,R.S)
Rural West Kassala (Kuliel area 12/10/2016)	E:36.22225^o^ N:15.50405^o^	200 sheep	23	3	Fever, diarrhea, oral erosions, nasal discharge	3	(N.S,W.B)
Kuliel area 12/10/2016	E:36.22225° N:15.50405°	230 sheep	8	20	Fever, Diarrhea, nasal discharge, cough	2	(R.S,W.B)
Rural Kassala (Fatou village 12/2/2017)	N:15.374091° E:36.381524°	120 sheep	14	4	Nasal discharge, grinting, diarrhea, oral erosion	2	(N.S,O.S,R.S)
Rural West Kassala (Kitralawaida area 10/11/2016)	E:36.18436^o^ N:15.47355^o^	380 sheep	37	6	Fever, nasal discharge, lacrimation, salivation,oral erosion, diarrhea	8	(Oc.S,N.S,O.S,R.S) and L.N
Rural West Kassala (Aledidat area 7/12/2016)	N:15.55470° E:35.94391°	300 sheep	20	4	Fever, abortion, nasal discharge, grinting, oral erosion, diarrhea	2	(O.S,N.S,R.S) and L.T
Rural West Kassala (Almarmadaib area 2/2/2017)	N:15.064989^o^ E:36.045371	500 sheep	30	25	Fever, lacrimation, Nasal discharge, diarrhea	5	(W.B)(N.S,F.S)
Rural West Kassala (Noora village 19/2/2017)	N:15.24129^o^ E:36.18719^o^	500 sheep	90	20	Fever, lacrimation, Nasal discharge, diarrhea,	8	(W.B,N.S,F.S)
Rural Kassala (Gllosit village22/2/2017)	N:15.32819^o^ E:36.15894	76 sheep	15 aborted	0	Abortion, nasal discharge, diarrhea	5	(W.B),N.S,R.S)
Rural Kassala (Kalhot area 22/2/2017)	N:15.26885^o^ E:36.16069	300 sheep	8	2	Fever, nasal discharge, oral erosion, bloody diarrhea	3	(W.B)(N.S,O.S,R.S)
Kassala (Elswagi Alshamaliya area 14/2/2017)	E:36.355586°N:15.470446°	80 sheep	10	4	Sudden death, fever, diarrhea, nasal discharge	2	(N.S,R.S)
		2876 total	9.5% ( morbidity)	3.2% (mortality)			

*O.S* Oral swab, *R.S* Rectal swab, *N.S* Nasal swab, *Oc*. *S* ocular swab, *L.N* Lymph nodes, *L.T* Lung tissue, *F.S* Fecal samples, *W.B* Whole blood

### Outbreaks data and sample collection

A total of 30 representative samples of 12 outbreaks were collected from sheep and goats with typical clinical signs of PPR in the febrile stage. Nasal, oral, ocular, and rectal swabs were collected using sterile swabs and preserved in transport media until analyzed. Blood in EDTA and fecal samples were also collected. Tissue samples including lymph nodes, lungs, intestines, spleens, and livers were aseptically collected from necropsied sheep. All samples were placed on ice in the laboratory, and then kept at -20 °C until examined. The data of suspected PPR outbreaks are demonstrated in Table 1.

### Sandwich ELISA

Sandwich ELISA was performed using a commercially available PPR antigen capture ELISA kit (IDvet, rue Louis Pasteur-Grabels-France). Thirty samples (two goats and 28 sheep) from the 12 PPR suspected outbreaks were screened for the presence of PPRV antigen (nucleoprotein (N)). The test was performed according to the manufacturer's instructions. The reaction optical density was read at 450 nm using a microplate reader (LINEAR, Barcelona, Spain).

### RNA extraction

Viral RNA was extracted from 22 samples using a commercial kit Qiamp Mini Kit (Qiagen, Germany) according to manufacturer’s instructions.

### One step RT-PCR

Twenty two samples which were positive by sELISA were tested for PPRV nucleic acid by one step RT-PCR amplification. The test was carried out using primer set directed to the conserved partial sequence of Ngene: (NP3) (Forward:5-TCTCGGAAATCGCCTCACAGACTG-3) and (NP4)(Reverse:5-CCTCCTCCTGGTCCTCCAGAATCT-3) [18]. RT-PCR for Ngene was carried out at 45 °C for 15 min to activate transcriptase enzyme. Initial denaturation was performed at 95 °C for 10 min, followed by 35 cycles of 94 °C for 30 s, 60 °C for 30 s, and 72 °C for 1 min, and a final extension step was performed at 72 °C for 10 min. For Fgene RT-PCR, 16 samples were selected from Ngene based RT-PCR positive samples using a primer set directed to the Fgene of PPRV: f1b (Forward:5-AGTACAAAAGATTGCTGATCACAGT-3) and f2d (Reverse:5-GGGTCTCGAAGGCTAGGCCCGAATA-3) [19]. RT-PCR for Fgene was carried out at 45 °C for 15 min to activate transcriptase enzyme, 95 °C for15 min as initial denaturation followed by35cycles of 95 °C for 1 min, 50 °C for1 min, and 72 °C for 2 min, and a final extension step was performed at 72 °C for 7 min using Biometra Thermocycler (Analytik Jena, Germany).

PCR products were subjected to electrophoresis in 2% agarose gel stained with ethidium bromide and visualized under ultraviolet (UV) light using a gel documentation system (Analytik Jena, Germany).

### Agreement between sELISA and N gene based RT-PCR

The agreement between Sandwich ELISA and Ngene based RT-PCR applied in this study using 12 samples was calculated as concordance percentage (%). Concordance percentage was calculated by division of the number of positive samples agreed by both sELISA and Ngene based RT-PCR by the total number of samples and multiplied by 100.

### Sequencing

Six Ngene based RT-PCR products ( three from border site (Kalhot village) and other three from e inner site (Noora village), were sent to Beijing Genomics Institute (BGI) (Beijing, China) for sequencing. The samples were sequenced using Sanger dideoxy Sequencing technology; both forward and reverse sequencing were performed on at least two independently generated PCR products. The sequencing results of six samples were subjected to Nucleotide-Nucleotide BLAST and Nucleotide-Protein BLAST tools in NCBI (www.ncbi.nlm.nih.gov) to confirm affiliation of the sequences to other PPRV isolates and translated sequences to PPRV nucleocapsid protein. Nucleotide sequences were submitted to the GenBank and get accession numbers: MG712792.1, MG712793.1, MG712794.1, MG712795.1, MG712796.1, and MG712797.1.

### Phylogenetic analysis

**Fig. 2:**
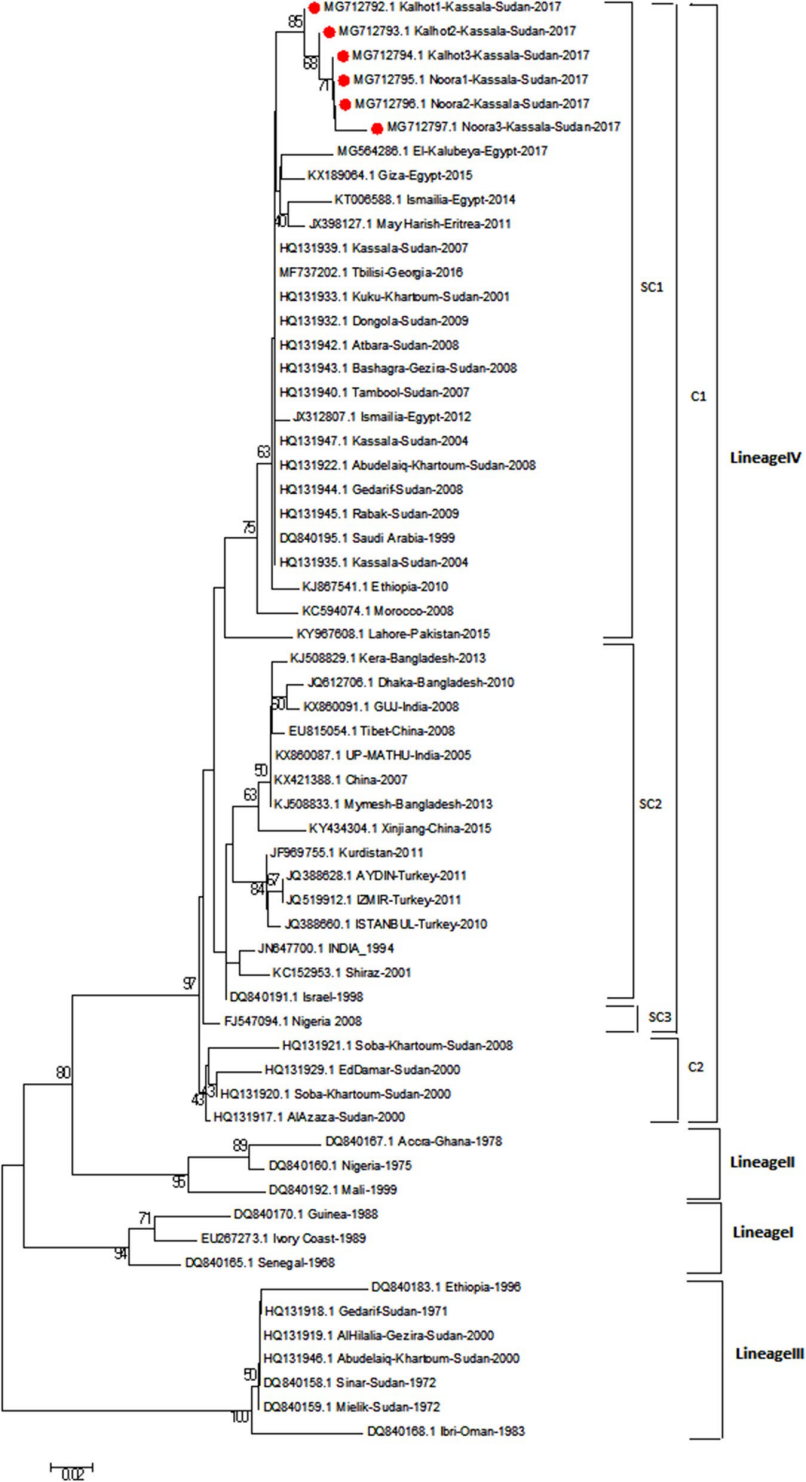
Neighbor-joining tree using partial N gene sequences (351 nucleotide bases) showed the relationships between PPRV isolates from Kassala State outbreaks (indicated by red circle) and NCBI submissions. The tree constructed by MEGA 6.0 program. The tree topology was evaluated by 1000 bootstrap replicates. Onlyvalues > 70% are shown

The sequences were retrieved from the GenBank database and used for the construction of a phylogenetic tree using MEGA 6.0 software [20]. The evolutionary history was inferred using the neighbor-joining method [21]. To present the identity relation between Kassala-2017isolates and other isolates from sub cluster1 in constructed tree (Fig. 2), we compared their partial sequences of N gene together. The sequences identities were calculated using MegAlign software (DNASTAR Lasergene, Madison, Wisconsin USA).

## Results

Among 30 samples tested by Sandwich ELISA, 22 (73.3%) were positive. Twenty two samples that were initially tested positive by sELISA were tested by Ngene based RT-PCR, and 17/22 samples (77.3%) were positive. Sixteen samples were selected from 17 samples tested positive by Ngene based RT-PCR, 7/16 (43.8%) were positive using Fgene based RT-PCR (Table 2). The length of the PCR amplicons was 351 bp for Ngene and 448 bp for Fgene. Twelve samples were used to compare sandwich ELISA and RT-PCR (Ngene), and out of them, 11 samples (animals) were positive for ELISA, nine for RT-PCR, and eight for both PCR and ELISA. This revealed an overall agreement (concordance) of 66.7% (8/12) between the two techniques (Table 3).

**Table 2:** Comparison of RT-PCR results for both genes that obtained from different samples in Kassala State, Eastern Sudan during 2015—2017

Sample Types	RT-PCR (N gene) *n* = 22		RT-PCR (F gene) *n* = 16	
	** + ve (%)**	**-ve**	** + ve (%)**	**-ve**
**Swab**	9 (81.8%)	2	2 (25%)	6
**Tissue**	3 (100%)	0	2 (100%)	0
**Fecal sample**	1 (100%)	0	0 (0%)	1
**Serum**	0 (0%)	1	0 (0%)	1
**Whole blood**	4 (100%)	0	3 (75%)	1
**Filter paper**	0	2	N/A	N/A
**Total**	**17 (77.27%)**	5	**7 (43.8%)**	9

**Table 3:** Cross-tabulation between sandwich ELISA and RT-PCR analysis results of 12 PPR clinical samples from Kassala State, Eastern Sudan during 2015 -2017

Technique	RT-PCR ( +)	RT-PCR (-)	Total	Concordance (%)
Sandwich ELISA ( +)	8	3	11	66.7%
Sandwich ELISA (-)	1	-	1	
Total	9	3	12	

The morbidity, mortality, and case fatality rates of the PPR outbreaks which are investigated in this study were 9.5% (273/2876), 3.2% (92/2876), and 33.7% (92/273) repectively. Sequences’ analysis of all six PPRV isolates in this study showed 98.3% -100% identity to each other.

**Fig. 3:**
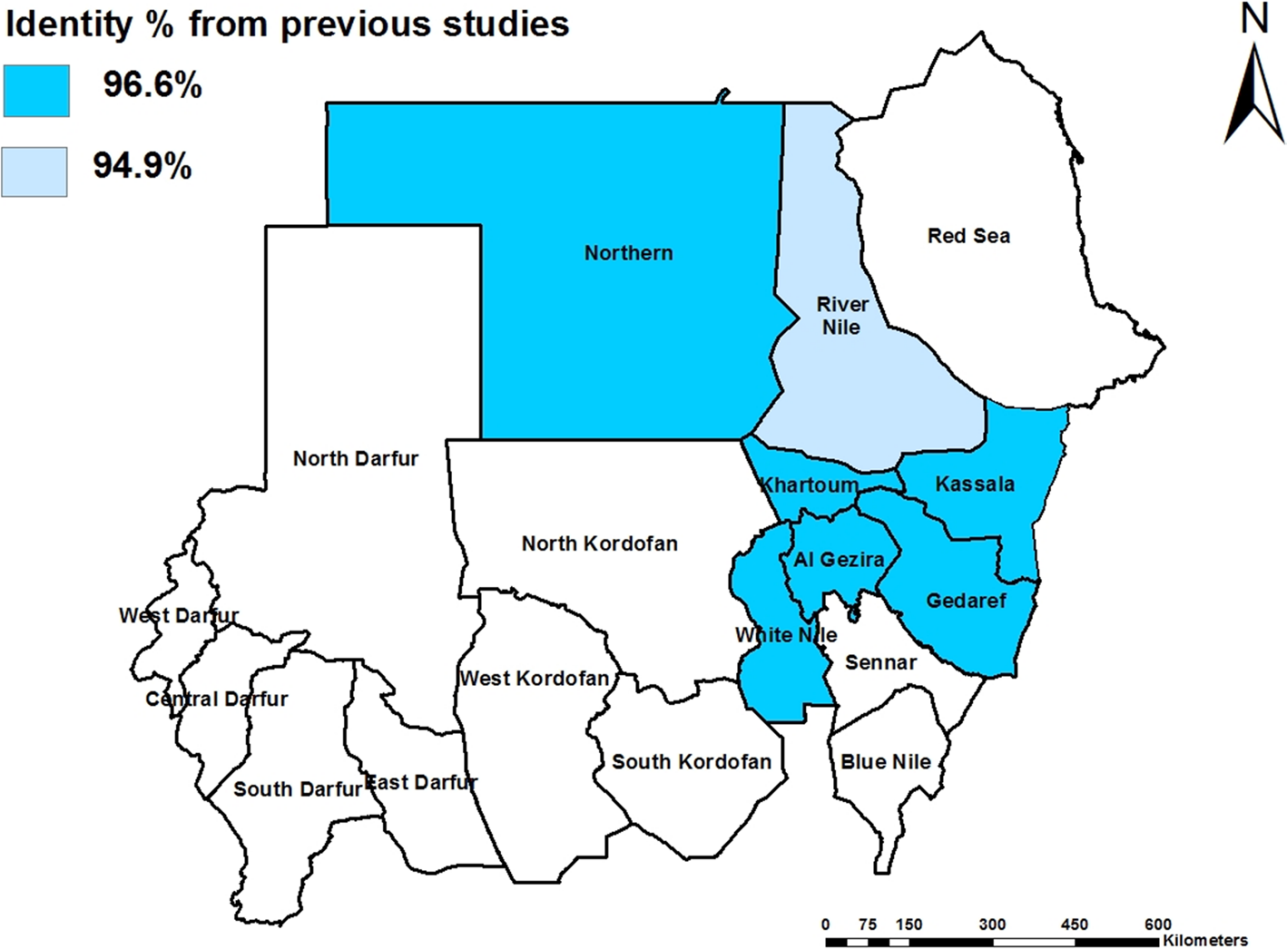
Nucleotide identity percent detected between Kassala PPRV strains (2017) and the other PPRV strains reported in the previous studies from Sudan

**Fig. 4:**
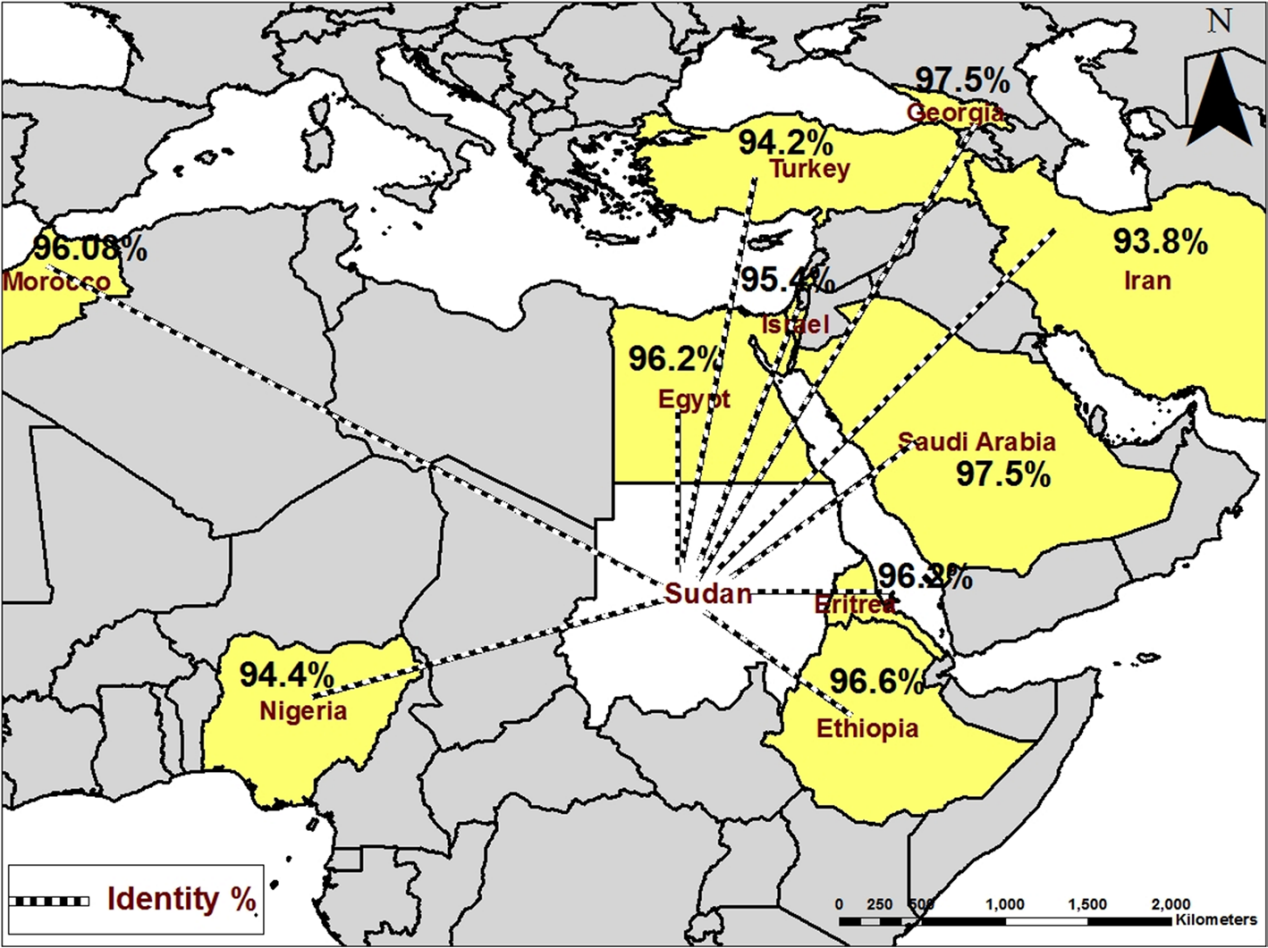
Nucleotide identity percent detected between Kassala PPRV strains (2017) and the other PPRV strains reported worldwide

Kassala-2017 isolates are clustered along with previous Kassala isolates, Egypt, Ethiopia, Eretria Georgia, Nigeria, Morocco, Saudi Arabia, Turkey, India, China, Bangladesh, Pakistan, Kurdistan, and Iran, while, other Sudanese isolates such as EdDamer, Alazaza and Soba are clustering together (Fig. 2). Nucleotide identity percentage between Kassala-2017 PPRV isolates and other PPRV isolates reported in the previous studies from Sudan was ranged between 94.9 and 96.6% (Fig. 3). Comparison of the nucleotide identity between Kassala-2017 PPRV isolates—and the other isolates in the subcluster1 of lineage IV indicated that the PPRV isolates from Kassala-2004 and 2007, Saudi Arabia-1999, and Georgia-2016 have an average nucleotide identity (97.5%) whereas an average (96.2%) of identity has been observed with Egypt isolates (2012, 2014, and 2015). In addition, Ethiopia-2010and Eretria-2011 PPRV isolates showed nucleotide identities were 96.6 and 96.2%, respectively, while Morocco -2008 isolate showed identity 96.08% (Fig. 4).

## Discussion

This study describes 12 suspected PPR outbreaks among unvaccinated sheep and goats flocks in four localities in Kassala State, Eastern Sudan during 2015- 2017. In Sudan, many outbreaks of PPR were investigated during 1999–2001 in Khartoum, Gezira, White Nile, North Kurdofan, and River Nile States [22]. In the course of PPR outbreaks surveys, classic clinical signs and symptoms were noticed including fever, diarrhea (bloody), nasal discharge, lacrimation, cough, grinting, oral erosions, salivation, and abortion. Similar manifestations were reported in previous studies in experimentally infected goats by Coucay Hymann et al. [23] and naturally infected goats by Ullah et al. [24]. The morbidity, mortality, and case fatality rates of the PPR outbreaks in this study were 9.5, 3.2%, and 33.7%, respectively. This is in line with Diallo [25] who recorded the morbidity and mortality rates due to PPR ranging from 0 to 90% which are based on local husbandry practices, breed, age, and other risk factors. In PPR outbreak in Punjab area of Pakistan, the morbidity rate was 31.7% (19/60), mortality rate was 3.3% (2/60), and case fatality rate was 10.5% [26]. In the present study, the presence of PPRV was proved by clinical signs, and antigen detection using Sandwich ELISA and RT-PCR techniques. The overall prevalence of PPRV herein reported was 73.3, 77.3, and 43.8% using Sandwich ELISA, RT-PCRs for Ngene and Fgene respectively. This agrees with Mahajan et al. [27] who reported that NP3 and NP4 primers for Ngene based RT-PCR showed the maximum sensitivity and specificity of PPRV detection in clinical samples compared Fgene primer based RT-PCR. This attributed to the first protein to be produced by PPRV. Ngene transcripts are produced before that of F-gene thus making N gene more suitable target for improving the sensitivity of RT-PCR for detection of PPRV from clinical samples [27]. Upon laboratory investigation, five samples were positive in Sandwich ELISA and were tested negative in Ngene based RT-PCR. This may be attributed to the presence of PCR inhibitors or viral RNA degradation (thermal degradation) in these samples.

Phylogenetic analysis of PPRV revealed that lineage I was limited to Western Africa and has not been reported since 1994 whereas, lineage II circulate in Western and central Africa. In addition, lineage III is restricted to East Africa and the Middle East [28]. Molecular typing conducted in this study indicated that Kassala-2017 isolates were clustered with lineage IV of PPRV. This is in accordance with Kwiatek et al. [29] who carried out molecular typing of enormous sampling of PPRV strains in Sudan during 2000–2009 and provided a strong evidence of the Asian lineage IV in Sudan. Similar results were reported by Khalafalla et al. [14] who characterized PPRV strains discovered among camels in Kassala State as lineage IV genotype. Moreover, the current study showed a genetic relationship between PPRV strains circulating in sheep in Kassala State, and PPRV strains characterized among camel in 2004 in the same state by Khalafalla et al. [14]. This finding is consistent with Kwiatek et al. [29] who reported that PPRV strains’ lineage IV was defined further by two sub-cluster: one included camel and goats’ isolates and some of sheep isolates, whereas the other contained only sheep isolates, and a finding suggests a genetic bias according to the host. The current study also indicated genetic relationship between PPRV strains circulating in sheep in Kassala State, Eastern Sudan, and PPRV strains in Eretria and Ethiopia. When comparing nucleotide sequences between Kassala- 2017 isolates and other isolates in the sub cluster1, it is found that there is high identity with these isolates that reach 100% (Fig. 3 and Fig. 4). These close similarities between Kassala- 2017 isolates and Eretria and Ethiopia isolates ensure transboundary circulation of PPRV due to free movement of animals searching for pastures across porous borders between Kassala, Eritrea, and Ethiopia. In addition there are live animals trading of camels and small ruminants for meat consumption between Kassala and Egypt through (Shalatain) aperture (Northern border of Sudan) in Red Sea State that it may be the main factor of similarity of viruses circulating in Kassala and Egypt. In addition there is a major trading activity between Saudi Arabia and Kassala State through airlines or through Swakin port on Red Sea..

## Conclusions

It could be concluded that PPRV belongs to the lineage IV genotype, which is responsible for outbreaks among sheep in Kassala State. In addition, the existence of the lineage IV in Kassala State may be due to spread of this lineage through transhumance and cross-border trade in small ruminants from neighboring countries where lineage IV is reported. Therefore, regular vaccination and regulation of animal movement between countries are important to establish crucial eradication program of PPR in Sudan.

## Data Availability

Data and materials are available upon request by the corresponding author.

## Abbreviations

PPRV: Peste des Petits Ruminants virus
sELISA: Sandwich enzyme-linked immunosorbent assay
RT-PCR: Transcription polymerase chain reaction
